# Hepatitis C virus subtype diversity and transmission clusters characteristics among drug users in Zhuhai, South China

**DOI:** 10.1186/s12879-024-09323-y

**Published:** 2024-04-29

**Authors:** Hongxia Li, Huitao Huang, Wenyan Huang, Man Du, Dongling Long, Guangxian Xu, Wenhua Mei, Kaisong Huang

**Affiliations:** 1 Zhuhai Center for Disease Control and Prevention, Zhuhai, Guangdong China; 2 School of Public Health, Jinan University, Guangzhou, Guangdong China; 3 Guangdong Provincial Key Laboratory of Medical Molecular Diagnostics, The First Dongguan Affiliated Hospital, Guangdong Medical University, Dongguan, Guangdong China

**Keywords:** Hepatitis C virus, Drug user, Phylogenetic tree, Molecular transmission network, High-risk factor

## Abstract

**Background:**

Hepatitis C virus (HCV) infection poses a major public health challenge globally, especially among injecting drug users. China has the world’s largest burden of HCV infections. However, little is known about the characteristics of transmission networks among drug user populations. This study aims to investigate the molecular epidemiology and transmission characteristics of HCV infections among drug users in Zhuhai, a bustling port city connecting Mainland China and its Special Administrative Regions.

**Methods:**

Participants enrolled in this study were drug users incarcerated at Zhuhai’s drug rehabilitation center in 2015. Their sociodemographic and behavioral information, including gender, promiscuity, drug use method, and so forth, was collected using a standardized questionnaire. Plasmas separated from venous blood were analyzed for HCV infection through ELISA and RT-PCR methods to detect anti-HCV antibodies and HCV RNA. The 5’UTR fragment of the HCV genome was amplified and further sequenced for subtype identifications and phylogenetic analysis. The phylogenetic tree was inferred using the Maximum Likelihood method based on the Tamura-Nei model, and the transmission cluster network was constructed using Cytoscape3.8.0 software with a threshold of 0.015. Binary logistic regression models were employed to assess the factors associated with HCV infection.

**Results:**

The overall prevalence of HCV infection among drug users was 44.37%, with approximately 19.69% appearing to clear the HCV virus successfully. Binary logistic regression analysis revealed that those aged over 40, engaging in injecting drug use, and being native residents were at heightened risk for HCV infection among drug user cohorts. The predominant HCV subtypes circulating among those drug users were 6a (60.26%), followed by 3b (16.7%), 3a (12.8%), 1b (6.41%) and 1a (3.85%), respectively. Molecular transmission network analysis unveiled the presence of six transmission clusters, with the largest propagation cluster consisting of 41 individuals infected with HCV subtype 6a. Furthermore, distinct transmission clusters involved eight individuals infected with subtype 3b and seven with subtype 3a were also observed.

**Conclusion:**

The genetic transmission networks revealed a complex transmission pattern among drug users in Zhuhai, emphasizing the imperative for a targeted and effective intervention strategy to mitigate HCV dissemination. These insights are pivotal for shaping future national policies on HCV screening, treatment, and prevention in port cities.

**Supplementary Information:**

The online version contains supplementary material available at 10.1186/s12879-024-09323-y.

## Introduction

Hepatitis C virus (HCV) infection can cause both acute and chronic hepatitis and is considered to be the leading cause of liver cirrhosis, hepatocellular carcinoma, and liver-related death, representing a significant public health issue globally [1]. The primary transmission routes of HCV include sharing contaminated needles and syringes for injecting drugs, transfusion of contaminated blood or blood products, engaging in unsafe sexual practices, and so forth [2, 3]. Following infections, only approximately one-third of individuals clear the virus spontaneously after the acute phase, while the rest generally develop into chronic infections and then are at high risk of progressing into cirrhosis and hepatocellular carcinoma [4]. According to endemic data from WHO, in 2019, an estimated 58 million people had chronic HCV infections globally [5]. Furthermore, it was reported that approximately 1.5 million new HCV infections and 290,000 HCV-associated deaths occurred annually [5]. In Mainland China, the overall prevalence rate of anti-HCV antibodies in the general population was around 0.91%, and this rate differed significantly across different geographical areas, ranging from 0.32 to 6.51% [6, 7].

HCV is a positive-sense, single-stranded RNA virus belonging to the Flaviviridae family [8]. The HCV genome is about 9.6 kb in size, coding three structure proteins and seven non-structure proteins arranged in the order 5’UTR-C-E1-E2-P7-NS2-NS3-NS4A-NS4B-NS5A-NS5B-3’UTR [8, 9]. Due to the lack of proof-reading activity by RNA polymerase, the HCV genome displays a high-level degree of genetic variability, characterized by a non-uniform distribution across the genome [10, 11]. Although the Core, E1, and NS5B regions were routinely utilized for HCV genotyping and subtyping, the 5’-UTR fragment, the most conserved region in the HCV genome, has recently emerged as an important target for the determination of HCV genotype and subtype across diverse populations [12–16]. To date, eight major HCV genotypes designated GT1 through GT8 have been identified worldwide, with further classification into up to one hundred subtypes [17, 18]. These HCV genotypes and subtypes exhibit distinct geographic distribution patterns globally. The subtypes 1a, 1b, 2a, and 3a are the most widely spread, particularly being highly prevalent in high-income countries [18]. In contrast, the GT5 was more prevalent in South Africa, while the GT6, like the 6a subtype, was mainly found in Southeast Asia, including Thailand, Vietnam, Myanmar, and China [18, 19]. The 1b and 2a are still the most prevalent subtypes in Mainland China, particularly in northern and central regions [7, 20, 21]. However, this situation is becoming increasingly complicated nowadays as more than 40% of newly detected cases in Southeast China were found to be GT6 and GT3, which was primarily linked to the widespread usage of addictive intravenous drugs [7, 13, 21].

Effectively halting the spread of HCV necessitates interrupting the transmission pathway between partners and accurately identifying the involved HCV genotypes for targeted treatments. The construction of HCV transmission networks can facilitate the identification of transmission clusters and the high-risk sociodemographic or behavioral factors associated with these transmissions. Traditionally, HCV transmission networks were primarily established through in-person interviews and partner tracing, which were labor-intensive and prone to biases. In recent years, molecular transmission network analysis based on genetic variations and evolutions has proven to be a powerful scientific tool for investigating the infectious disease transmission network and identifying associated genotypes for tailored treatment, particularly in the context of HIV transmission [22, 23]. This approach recently has also been successfully applied to delineate HCV transmission networks, demonstrating its efficacy and convenience in accurately identifying underlying links and reflecting actual relationships among infected partners across diverse global populations [3, 14, 24–26].

Zhuhai, one of China’s major port cities, uniquely served as the sole conduit connecting Mainland China to its Special Administrative regions of Hongkong and Macau. Cross-border customs clearance volumes between Zhuhai and these regions are exceptionally high, with the Gongbei Port processing over 300,000 daily crossings. Moreover, this region has a significantly high prevalence of drug use, further making HCV disease management in Zhuhai even more complicated and challenging. To elucidate the HCV transmission dynamic in this complex setting, we conducted a comprehensive HCV investigation among drug users who were incarcerated in the drug rehabilitation center of Zhuhai. Risk factors associated with HCV dissemination among these individuals were explored and delineated. By analyzing the 5 ‘UTR sequences, we constructed an HCV phylogenetic tree and molecular transmission networks with the incorporation of epidemiological data. These results will inform targeted interventions by health authorities to curb HCV transmissions in this major Chinese transportation hub.

## Materials and methods

### Study participants

Data and specimens for this cross-sectional survey were obtained from 435 participants enrolled in the national sentinel surveillance program for anti-HCV in Zhuhai in 2015. All participants were drug users with a history of continuous use of heroin, cocaine, methamphetamine, or other psychotropic substances known to induce non-medical addiction and were incarcerated in the drug rehabilitation center of Zhuhai. It’s noteworthy that identified drug users in Zhuhai without severe health conditions, such as HIV infections, are typically subjected to compulsory incarceration and rehabilitation at this facility. All drug users incarcerated at this center during the months of April and October 2015, respectively, were recruited and included in this study, excluding individuals who participated more than once. Prior to enrollment, all participants provided informed consent by signing consent forms, indicating their voluntary participation in this research.

### Specimens and testing for HCV infections

A total of 435 anticoagulant venous blood samples were collected from the study participants in 2015. Each participant also completed a National standardized questionnaire capturing sociodemographic and behavioral information, including gender, age, promiscuity, educational level, marital status, place of residence, and drug use method. Upon collection, the anticoagulant blood samples were promptly transported to the laboratory, and the plasma was isolated by centrifuging at 1900 g at 4 °C for 10 min. All isolated plasma was tested for anti-HCV antibodies using the ELISA kit (Wantai, China) according to the instructions provided by the National standard. The remaining plasmas were preserved at -80 ˚C for future use. In 2022, RNA extraction from the plasma was carried out using the QIAamp Viral RNA Mini Kit (Qiagen, China) following the manufacturer’s instructions. Subsequently, HCV RNA detection was conducted using a real-time quantitative PCR approach with the TaqMan Universal Master Mix II (UNG included) Kit (AppliedBiosystems, China) following the National instructions. The specific primer set and probe sequence used for the PCR were as follows: forward primer (CGGGAGAGCCATAGTGGT), reverse primer (CAAGCACCCTATCAGGCA), and probe (FAM-CAA GGCCTTTCGCGACCCAA -BHQ1). In this study, HCV infections encompass current infections and past infections. The past infections indicate that the HCV virus has either been spontaneously cleared or successfully treated. Current infections were identified by the positive HCV RNA test result, while past infections were characterized by being anti-HCV antibody positive but negative for HCV RNA. Acute hepatitis C virus (HCV) infection is defined as acquiring the virus less than six months ago, regardless of clinical signs or symptoms. In contrast, chronic HCV infection indicates having the virus for over six months.

### PCR amplification and sequencing

The HCV RNA-positive samples were further subjected to genome amplification and sequencing analysis. In brief, the isolated RNA was firstly reverse transcribed into first-strand cDNA using the Takara One Step RNA PCR Kit (AML) kit. Subsequently, a 401-bp segment at the 5’UTR of the HCV genome was amplified using 5’UTR F/R primers. The amplified products were visualized by gel electrophoresis and then purified for Sanger sequencing (Huirui. China). The sequences of forward and reverse primer were F-TGGGGATCCCGTATGATACCCGCTGCTTTGA and R-GGCGGAATTCCTGGTCATAGCCTCCGTGAA, respectively.

### HCV genotypes analysis and phylogenetic tree reconstruction

The raw sequences obtained were processed and cleaned using Chromas software version 2.6.5. Subsequently, the sequences were corrected using BioEdit version 7.0 and aligned to the GenBank database using the NCBI online BLAST tool (http://www.ncbi.nlm.nih.gov/genbank/) for genotype and subtype determination. Following this, all sequences were aligned using ClustalW and standardized to a uniform length using MEGA software version 7.0.

The reference sequences DQ155490.2, KJ678421.1, KU180720.1, FJ696531.1, DQ640353.1, and KY620855.1 from GenBank were selected because they were genetically close to the subtypes in this study. The phylogenetic tree was constructed in MEGA 7.0 with the Maximum Likelihood method and the Tamura-Nei model. The resulting phylogenetic tree was further visualized and edited using the iTOL online platform.

### HCV molecular transmission network reconstruction

Pairwise genetic distances among the sequences were calculated using the TN93 model implemented in the HyPhy2.2.4 software. By examining the total number of sequences and propagation clusters within the transmission networks across a range of thresholds, it was observed that the number of propagation clusters and sequences included in networks was the largest when the threshold was set to 0.015. Therefore, a molecular transmission network was constructed using Cytoscape3.8.0 software with a threshold of 0.015 to identify potential transmission relationships. The degree in the molecular transmission network represents the number of edge connections between nodes. The access rate is the percentage of the total number of sequences that enter the molecular transmission network.

### Statistical analyses

Statistical analyses were performed using SPSS 27.0. Categorical variables were expressed as frequency and percentages, and the chi-square test was used to compare infection differences among distinct variable groups. Binary logistic regression models were further used to analyze the influencing factors associated with HCV infection. A *p*-value less than 0.05 was considered statistically significant.

## Results

### Sampled populations and HCV prevalence

All identified drug users in Zhuhai were compulsorily incarcerated and rehabilitated in the city’s drug rehabilitation center. In 2015, a total of 435 drug users from this facility were enrolled in this study. Of these participants, 391(89.9%) were male, and 44(10.1%) were female. The age distribution of the participants ranged from 17 to 61 years old, with a mean age of 34.5 years and a median age of 35 years. Among the 435 participants, 51(11.7%) individuals were either divorced or widowed, 282(64.8%) were local residents, 381(87.6%) denied engaging in promiscuous sexual behavior, and 280(64.4%) denied injecting drug use (Table 1 and Supplementary file 1).

**Table 1:** Analysis of the associated factors of HCV infection among drug abusers in Zhuhai

Variable		Counts (Frequency %)	only anti-HCV (+)** (Counts)	anti-HCV (+) and HCV RNA (+)** (Counts)	only HCV RNA (+)** (Counts)	HCV (+)**				
						case (infection rate%)	BLR*** (*P* value)	OR (95%CI)		
**Total**		435(100)	38	147	8	193 (44.37)				
Gender*							0.109			
Male		391(89.9) (Injected drugs: *n* = 151(38.62%))	33	140	7	180(46.04)				
Female		44(10.1) (Injected drugs: *n* = 4(9.09%))	5	7	1	13(29.55)				
Age*							0.001			
Mean	35									
Median	35									
Minimum	17									
Maximum	61									
Std.Deviation	8									
≥ 40		106(24.4). (Injected drugs: *n* = 55(51.8%))	18	56	1	75(70.75)	0.000 (≥ 40 vs.31~40 )	6.858 (3.021–15.567)		
31~40		178(40.9). (Injected drugs: *n* = 87(48.8%))	15	78	0	93(52.25)	0.211 (31~40 vs. ≤30 )			
≤ 30		151(34.7). (Injected drugs: *n* = 13(8.6%))	5	13	7	25(16.56)				
**Sex promiscuity**										
yes		54(12.4)	5	19	0	24(44.44)				
No		381(87.6)	33	128	8	169(44.36)				
Injecting drug use*							0.000	48.881 (22.765–104.960)		
Yes		155(35.6)	28	113	1	142(91.61)				
No		280(64.4)	10	34	7	51(18.21)				
**Educational level**										
Untaught		12(2.8)	2	6	0	8(66.67)				
Primary school		99(22.8)	5	41	2	48(48.48)				
Junior high school		239(54.9)	25	77	3	105(43.93)				
High school or technical secondary school		73(16.8)	5	20	2	27(36.99)				
College and above		12(2.8)	1	3	1	5(41.67)				
Marital status*							0.166			
Unmarried		176(40.5) (Injected drugs: *n* = 41(23.30%))	10	36	3	49(27.84)				
Married		153(35.2) (Injected drugs: *n* = 75(49.02%))	16	70	2	88(57.52)				
Cohabit		55(12.6) (Injected drugs: *n* = 13(23.64%))	4	19	1	24(43.64)				
Divorced or widowed		51(11.7) (Injected drugs: *n* = 26(50.98%))	8	22	2	32(62.73)				
Place of residence *							0.000	3.625 (1.877–7.001)		
native. (Guangdong)		282(64.8) (Injected drugs: *n* = 129(51.8%))	28	125	4	157(55.67)				
outsider		153(35.2) (Injected drugs: *n* = 26(51.8%))	10	22	4	36(23.53)				

*Infection differences among the groups of gender, age, marital status, injecting drug use, and place of residence were compared by Chi-Square tests, and all showed statistical significance (*P* < 0.05)

** anti-HCV (+) = Positive anti-HCV, HCV RNA (+) = Positive HCV RNA, HCV (+) = Positive HCV RNA and/or Positive anti-HCV

***BLR stands for Binary Logistic Regression

Of the 435 anticoagulant venous blood samples collected, 147 tested positive for both HCV RNA and anti-HCV antibodies, eight were positive for HCV RNA but negative for anti-HCV antibodies, 38 were negative for HCV RNA but positive for anti-HCV antibodies, and 242 were negative for both HCV RNA and anti-HCV antibodies. The overall HCV infection rate was 44.37% (193/435), comprising 147 dually positive samples, eight samples positive solely for HCV RNA, and 38 positive solely for anti-HCV antibodies. (Table 1). Since most HCV-infected individuals could not provide a definitive infection date, accurate differentiation between acute and chronic infection status was not feasible. Among the 193 HCV-infected drug users, only 19.69% (38/193) appeared to have successfully cleared the HCV virus based on an anti-HCV antibody positive but HCV RNA negative profile. The eight individuals who tested positive for HCV RNA yet negative for anti-HCV antibodies likely experienced a recent HCV infection or were immunocompromised. Of these 193 HCV-infected persons, 180 were male, 150 were local residents, and 142 reported injecting drug users (Table 1).

### Influencing factors of HCV infection

In order to evaluate the risk of sociodemographic and behavioral factors associated with HCV infections among drug users in Zhuhai Port City, a chi-square testing analysis was employed to compare infection differences across different variable groups, including gender, age, promiscuity, educational level, marital status, place of residence, and drug use and so forth. The results revealed that the infection rate among groups of the variables of gender, age, marital status, injecting drug use, and place of residence were statistically significant. Binary logistic regression analysis further substantiated that HCV infection can be influenced by age (*P* = 0.000 < 0.05, OR = 6.858, 95%CI: 3.021–15.567), injecting drug use (*P* = 0.000 < 0.05, OR = 48.881, 95%CI: 22.765–104.960), and place of residence (*P* = 0.005 < 0.05, OR = 3.625, 95%CI: 1.877–7.001). Conversely, no significant difference was observed in HCV infections between those with or without promiscuous sexual behavior or among individuals with different educational levels.

### HCV genetic analysis

Among the 155 HCV RNA-positive specimens, the 5’-UTR fragments of around 110 samples were successfully amplified and purified for sequencing. After excluding samples with failed or poor sequencing quality, a total of 78 valid sequences were eventually obtained for subtype determination and phylogenetic analysis. The predominant subtype identified among these HCV specimens were 6a (60.26%, *n* = 47), followed by 3b (16.7%, *n* = 13), 3a (12.8%, *n* = 10), 1b (6.41%, *n* = 5) and 1a (3.85%, *n* = 3), respectively. Notably, the majority of these 78 individuals were local residents (87.2%), and most denied engaging in promiscuous sexual behaviors (91.0%) but reported a history of injecting drug use (84.%). Furthermore, all of them were also positive for anti-HCV antibody testing.

**Fig. 1:**
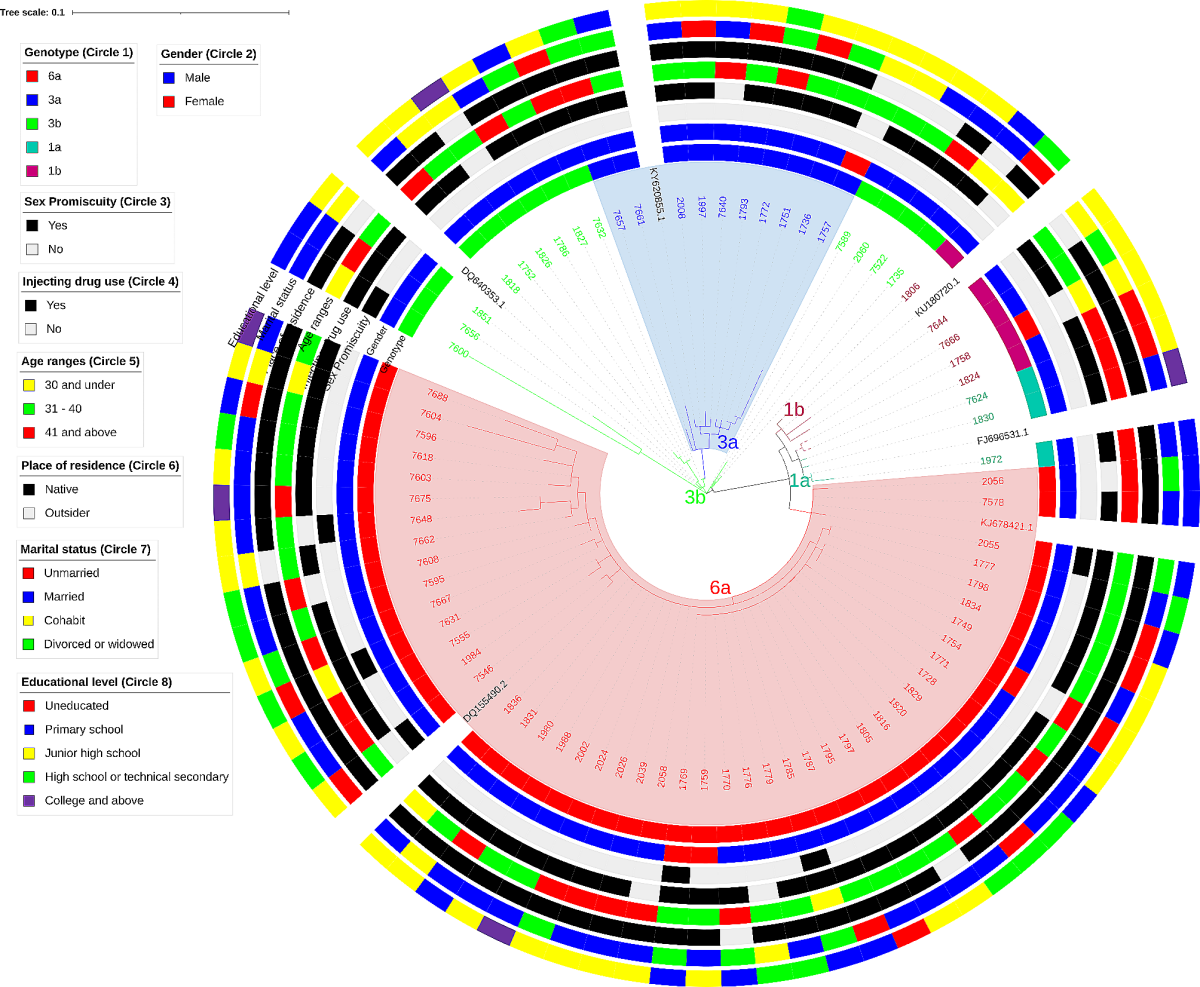
Maximum-likelihood phylogenetic tree of the HCV 5’UTR sequences from 6 reference sequences and 78 drug users in Zhuhai, China. The HCV genotypes were labeled in different colors. The outside circles provide sociodemographic and behavioral information on these individuals

The phylogenetic tree, constructed using the 78 sequences from this study and six reference sequences, revealed a high degree of genetic diversity among these sequences, particularly within the subtype 3b category, which comprised four distinct branches. In contrast, the ten sequences of subtype 3a clustered together and were genetically close to the subtype of 3b (Fig. 1). The sequences of subtypes 1a and 1b category showed the least distinction and were genetically closer to the subtype 6a family instead. Notably, the 47 sequences of subtype 6a formed the largest group in this phylogenetic tree and clustered separately from other subtype sequences, although two sequences within this subtype (Seq No. 2055 and 2056) diverged to form two distinct clades with the rest of the sequences (Fig. 1).

### Molecular propagation network analysis

**Fig. 2:**
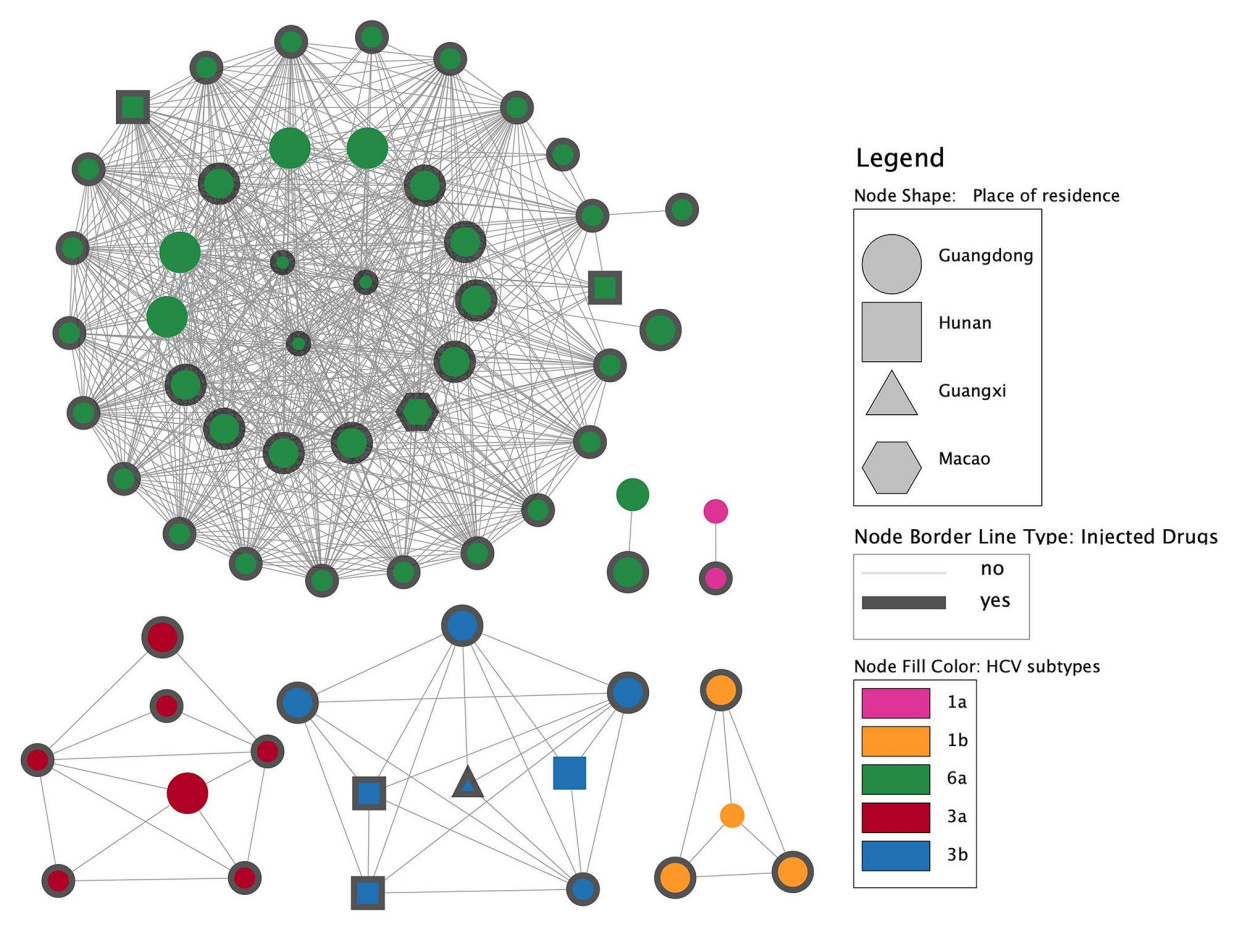
Hepatitis C virus molecular transmission network of drug users in Zhuhai, China. Six transmission clusters were identified, with the number of sequences per cluster varying from 2 to 41. Each node represents a person, and the line between the two nodes indicates a transmission line. The fill color, shape, borderline type, and size of each node indicate the HCV subtype, residence place, history of injecting drug use, and age details of the individual, respectively. The three diameter levels of the node, starting from the smallest, represent the age range less than 30, 31–40, and over 40, respectively

The molecular propagation network analysis was conducted using a threshold of 0.015, resulting in 64 sequences entering the molecular propagation network with an access rate of 82.05% (64/78) and degrees ranging from 1 to 37. The network revealed the presence of six distinct clusters, with the number of sequences per cluster varying from 2 to 41. Within these clusters, each node represents a person, and the line between 2 linked persons indicates a transmission line (forming one link). The largest propagation cluster in the molecular transmission network consisted of 41 individuals infected with HCV subtype 6a (Fig. 2). Following that, there were clusters with eight individuals infected with subtype 3b, seven with subtype 3a, four with subtype 1b, two with subtype 1a, and another two with subtype 6a, respectively. Among the 64 individuals in the network, 57 were native to Guangdong Province (registered in Guangdong), and only seven were from other places, showing a statistically significant difference (Supplementary Table 1). Moreover, out of these 64 individuals, 60 were male, and 55 individuals were identified as injecting drug users. Of the five people entering the transmission network and acknowledging a history of sex promiscuity, they all belong to the largest propagation cluster infected by subtype 6a. Notably, of the nine drug users who entered the transmission network but denied injecting drug use, eight of them also denied a history of sex promiscuity (Supplementary Table 1).

## Discussion

This study characterized the epidemiology and molecular transmission characteristics of HCV infections among drug users in Zhuhai, a port city in southern China with abundant tourism resources due to connecting Mainland China with its Special Administrative regions. We observed dynamic HCV infections among drug users in Zhuhai predominated by the subtypes of 6a, 3a, and 3b, and these infections also lead to the primary transmission clusters among these drug users. In addition, the sociodemographic and behavioral factors of age over 40, injecting drug use, and being native residents were high-risk factors for HCV infection among these drug user populations.

The findings revealed an overall HCV infection rate of 44.37% among drug users in Zhuhai, which is similar to the reported average rate of Guangdong Province during the first decade of the 21st century (44.63%) [27]. This similarity suggests that the intervention strategies implemented by relevant authorities in Zhuhai may not have been effective in recent years. Among these infected drug users, only 19.69% successfully cleared up their infections, a clearance rate significantly lower than the approximately 30% reported in general populations in previous studies [4, 28, 29]. Further researches are warranted to elucidate the potential relationship between drug abuse and immune impairment affecting HCV clearance.

Previous research has consistently reported that the high prevalence of HCV infection among drug users was associated with sharing syringes [30]. In our study, the binary logistic regression analysis confirmed that injecting drug use can significantly and substantially increase the risk of HCV infections. Moreover, our findings revealed that older age groups (above 40 years) were associated with a higher risk of HCV infections. This association may be attributed to a more extended history of drug abuse, leading to more severe immune impairment and an increased likelihood of chronic infections. Binary logistic regression analysis also demonstrated that local drug users had a significantly higher risk compared to non-locals. This phenomenon is likely attributable to the city’s port characteristics, which can provide local residents with more opportunities for drug exposure.

Hepatitis C Virus exhibits a broad global distribution, with significant regional variations in genotype prevalence [17, 18]. China has recorded six genotypes and 24 subtypes to date, with the subtypes 1b and 2a being the most predominant [9, 31]. In our study, we found that the predominant HCV subtypes among drug users in Zhuhai, a port city in Guangdong Province in China, were 6a (60.26%), followed by 3b (16.7%) and 3a (12.8%), respectively. This predominance of the 6a subtype in Zhuhai is consistent with previous reports, as 6a was mainly prevalent in South China, like the Guangdong and Guangxi provinces [9, 31]. The genotype GT3 (3a and 3b) primarily circulated in Southwest China, like Yunnan province, and East Asian countries, where drug smuggling and intravenous drug abuse were prevalent [9, 31–33]. Surprisingly, the prevalence of genotype GT3 was also remarkably high among drug users in Zhuhai, reaching approximately 30%. This finding suggests that the transmission of HCV subtypes 3a and 3b may have occurred through drug trafficking activities from Yunnan province into cities in South China during these years, like Zhuhai.

Molecular transmission network analysis offers a more precise method for identifying underlying connections and reflecting the real transmission relationships between infected individuals, especially in cases where individuals are reluctant to disclose their partner’s information due to self-protection concerns. In our study, the largest propagation cluster identified consisted of 41 individuals infected with HCV subtype 6a. This discovery can guide authorities in Zhuhai in implementing tailored intervention measures and treatments specific to this subtype among drug users. Among the 64 drug users entering the molecular transmission network, 57 were local residents (Supplementary Table 1), indicating that drug users are prone to form partnerships with individuals from the same region, possibly due to shared resources, living circumstances, and familiarity.

Interestingly, among the 64 drug users in the molecular transmission network, nine individuals denied engaging in injecting drug use, and eight of these nine people also denied any history of sexual promiscuity. However, the molecular transmission network analysis revealed that seven out of these nine individuals may had partnerships with at least three or more drug users. This highlights the challenges of accurately tracing partners among drug users using conventional questionnaire methods, as individuals may withhold information about their shared injecting behaviors, drug sources, and their injection partners due to shame or fear of revenge. Therefore, using molecular transmission networks to investigate HCV dissemination in conservative populations can help overcome the limitations of traditional contact tracing methods, which are often influenced by recall bias and social expectation deviation.

### Limitations of the study

There are three main limitations in this study. First, the sample size is relatively small, which could impact the statistical outcomes and the insights into the molecular transmission network. Second, we were unable to distinguish the individual initially infected from those involved in subsequent transmission events. An approach that combines consecutive sampling over a longer duration and the analysis of the HVR1 fragment could offer us more details of the transmission network, including the source. Third, some of the HCV RNA may be degraded due to the long-term storage of the samples, which likely affects the positive rate of HCV-RNA testing and subsequent PCR amplifications.

## Conclusion

In conclusion, our study identified injecting drug use, being over 40 years old, and being a native resident as high-risk factors for HCV infection among drug users in Zhuhai. The predominant HCV subtypes among those drug users were 6a (60.26%), 3b (16.7%), and 3a (12.8%), respectively. Using molecular transmission networks can allow us to assess the dissemination risk of HCV and identify the transmission cluster, thus providing a more accurate basis for preventing and controlling HCV infection.

### Electronic supplementary material

Below is the link to the electronic supplementary material.

Supplementary Material 1

Supplementary Material 2

## Data Availability

All data generated or analyzed during this study are included in this published article [and its supplementary information files].

## Abbreviations

5’UTR: 5’ Untranslated Region
CI: Confidence Interval
ELISA: Enzyme Linked Immunosorbent Assay
HCV: Hepatitis C virus
HIV: Human immunodeficiency virus
OR: Odds Ratio
RT-PCR: Reverse transcription polymerase chain reaction
