# Supplementary material for: Hepatitis C virus subtype diversity and transmission clusters characteristics among drug users in Zhuhai, South China
Source: BMC Infect Dis. 2024 Apr 29;24:451. doi: 10.1186/s12879-024-09323-y (PMC11057121; doi:10.1186/s12879-024-09323-y)
Supplement: Supplementary file 2 — Supplementary Material 2 [file 12879_2024_9323_MOESM2_ESM.docx]

| **Supplementary table 1. The sociodemographic features and drug use behavior of individuals entering the molecular transmission network** | | | | |
| --- | --- | --- | --- | --- |
|  | **Overall (%)** | **Not in network (%)** | **In network (%)** | **Chi-Square (P value)** |
| **Total** | 78(100) | 14(17.9) | 64(82.1) |  |
| **Gender** |  |  |  | 0.676 |
| Male | 73(93.6) | 13(16.7) | 60(76.9) |  |
| Female | 5(6.4) | 1(1.3) | 4(5.1) |  |
| **Age** |  |  |  | 0.504 |
| ≤30 | 8(10.3) | 2(2.6) | 6(7.7) |  |
| 31～40 | 42(53.8) | 8(10.3) | 34(43.6) |  |
| >40 | 28(35.9) | 4(5.1) | 24(30.8) |  |
| **Sex promiscuity** |  |  |  | 0.139 |
| yes | 7(9.0) | 2(2.6) | 5(6.4) |  |
| No | 71(91.0) | 12(15.4) | 59(75.6) |  |
| **Injecting drug use** |  |  |  | 0.328 |
| Yes | 66(84.6) | 11(14.1) | 55(70.5) |  |
| No | 12(15.4) | 3(3.8) | 9(11.5) |  |
| **Educational level** |  |  |  | 0.286 |
| Untaught | 1(1.3) | 0(0.0) | 1(1.3) |  |
| Primary school | 17(21.8) | 4(5.1) | 13(16.7) |  |
| Junior high school | 41(52.7) | 7(9.0) | 34(43.6) |  |
| High school or technical secondary school | 14(17.9) | 2(2.6) | 12(15.4) |  |
| College and above | 5(6.4) | 1(1.3) | 4(5.1) |  |
| **Marital status** |  |  |  | 0.098 |
| Unmarried | 15(19.2) | 1(1.3) | 14(17.9) |  |
| Married | 39(50.0) | 6(7.7) | 33(42.3) |  |
| Cohabit | 11(14.1) | 5(6.4) | 6(7.7) |  |
| Divorced or widowed | 13(16.7) | 2(2.6) | 11(14.1) |  |
| **Place of residence** |  |  |  | 0.005 |
| Guangdong(native) | 68(87.2) | 11(14.1) | 57(73.1) |  |
| Hunan | 8(10.3) | 3(3.8) | 5(6.4) |  |
| Guangxi | 1(1.3) | 0(0.0) | 1(1.3) |  |
| Macao | 1(1.3) | 0(0.0) | 1(1.3) |  |
